# Acute phase proteins in canine and feline fetal fluids during the second half of gestation

**DOI:** 10.1016/j.vas.2024.100415

**Published:** 2024-11-27

**Authors:** Hossein Sahraei, Asghar Mogheiseh, Fatemeh Doudmani, Nasrin Kazemipour, Saeed Nazifi

**Affiliations:** a Department of Clinical Sciences, School of Veterinary Medicine, Shiraz University, Shiraz, Fars, Iran; b Department of Basic Sciences, School of Veterinary Medicine, Shiraz University, Shiraz, Fars, Iran

**Keywords:** Acute phase proteins, Pregnancy, Fetus, Amniotic, Allantoic

## Abstract

The present study aimed to evaluate the acute phase proteins (serum amyloid A, alpha-1 acid glycoprotein, and C-reactive protein) profile in canine and feline fetal fluids during the second half of gestation. Seventeen pregnant domestic short-hair queens and 19 pregnant mixed-breed bitches were enrolled. Animals were divided into three groups according to their gestational age (30–39, 40–49, and 50–60 days). After ovariohysterectomy and sample collection, the concentration of the mentioned APPs was measured by the ELISA method. There was an increasing trend in the concentration of APPs in canine fetal fluids alongside increasing pregnancy age, but these changes were not statistically significant. Canine amniotic SAA concentration was significantly higher than allantoic. The highest concentration of SAA and CRP in the feline fetal fluids was observed at days 40–49, and the maximum concentration of AGP was observed at days 50–60. For the first time, the current study investigated the APPs profile in canine and feline fetal fluids during pregnancy. The maximum concentration of APPs was observed at the end of canine pregnancy and at days 40–49 of feline pregnancy.

## Highlights

• The acute phase proteins were determined in canine and feline fetal fluids.
• Canine amniotic SAA concentration was significantly higher than allantoic.
• The highest concentration of SAA and CRP in the feline fetal fluids was observed at days 40–49.
• The maximum concentration of APPs was observed at the end of canine pregnancy.

**List of abbreviations**
SAA: Serum amyloid A
CRP: C reactive protein
APPs: Acute phase proteins
AGP: Alpha-1-acid glycoprotein
PTX3: Pentraxin3
IAI: Intra-amniotic inflammatory
PROM: Preterm pre-labor rupture of membranes

## 1 Introduction

Healthy growth and development during pregnancy prepare the fetus for the challenging extra uterine environment, so clinical assessment is necessary to ensure a normal pregnancy. For efficient clinical assessment, it is necessary to have comprehensive knowledge of pregnancy physiology and the changes that occur during gestation and to detect biomarkers that can be used to identify pathological conditions at each stage of pregnancy (Vannucchi et al., 2002).

Acute-phase proteins (APPs) mediate the acute-phase reaction (APR) to local or systemic stimuli and maintain homeostasis shortly after stress, inflammation, trauma, surgery, neoplasia, and infection (Eckersall & Bell, 2010). Following these conditions, proinflammatory cytokines such as IL-6, IL-1, and TNF-α provoke the release of positive APPs, including serum amyloid A (SAA), alpha-1 acid glycoprotein (AGP), complement component3, C-reactive protein (CRP) and hemopexin into the blood (Baumann & Gauldie, 1994). Major APPs in cats are AGP and SAA; in dogs, CRP and SAA are the major ones (Cerón et al., 2005). Measuring the concentration of positive APPs has been used as a diagnostic-prognostic method, part of screening tests, and for monitoring treatment/drug response (Rosa & Mestrinho, 2019b). The use of serum concentration of APPs in the early diagnosis of canine pregnancy has been reported by different researchers (Evans & Anderton, 1992; Kuribayashi et al., 2003; Vannucchi et al., 2002); however, it should be considered that APPs are susceptible to inflammation caused by various agents, but they are not specific markers for inflammation due to specific causes. Therefore, before evaluating APPs for pregnancy diagnosis, checking the animal's general health condition is necessary. Nevertheless, these biomarkers can be utilized to monitor the health of pregnant dogs and diagnose any risks related to embryonic and fetal losses (Ulutas et al., 2009).

The mother and fetus each play a unique role in the formation of fetal fluids, including allantoic and amniotic fluids. It has been reported that changes in the composition of these fluids reflect changes occurring during intrauterine development, so evaluation of these fluids and their components can be used as an efficient method for pregnancy clinical assessment, as has been utilized in human medicine and more recently in dogs and cats (Bigliardi et al., 2022; Fresno et al., 2012; Veronesi et al., 2018). The concentration of CRP, serum amyloid P, and pentraxin3 (PTX3) in amniotic fluid has been found to contribute to intra-amniotic inflammatory (IAI) complications in cases of preterm pre-labor rupture of membranes (PROM) in humans. Among these markers, PTX3 has been identified as the most reliable diagnostic marker for detecting these complications (Musilova et al., 2018).

Recent advancements in canine and feline perinatology highlight the importance of the prenatal period for fetal growth and neonatal survival. Understanding the physiology of this period is crucial for identifying normal versus pathological pregnancies, enabling early detection of neonates requiring special care (Veronesi et al., 2018). Fetal fluids play a key role in providing a protective environment for development, with amniotic fluid containing secretions from various fetal systems and allantoic fluid primarily originating from the fetal urinary system (Bigliardi et al., 2022). The composition of these fluids can be affected by maternal and fetal conditions, such as stress and nutrition (Bigliardi et al., 2022; Musilova et al., 2018). Acute phase proteins (APPs) are critical for immune response and homeostasis, with positive APPs like CRP and SAA significantly increasing during inflammation. Notably, differences in APP concentrations between pregnant and non-pregnant bitches can be observed, particularly around the time of implantation (Vannucchi et al., 2002). Research in human amniotic fluid has identified antimicrobial agents and inflammatory markers that are crucial for diagnosing intra-amniotic infections (Meloni, 2015). The presence of these components suggests that similar studies in canine and feline fetal fluids could enhance diagnostic techniques and improve neonatal care.

Studies on pregnant women revealed that CRP is a physiological component of amniotic fluid (Borna et al., 2009). To date, no study has investigated the presence of APPs in canine and feline fetal fluids, so this study set out to (1) investigate the presence and changes of CRP, SAA, and AGP in amniotic and allantoic fluids during days 30 to 60 of pregnancy in dogs and cats; and (2) determine the normal values of acute phase proteins in fetal fluids. This will enable us to conduct comprehensive research on pathological pregnancies to identify potential biomarkers. The ultimate goal is to develop effective screening tests and provide efficient medical assistance for high-risk pregnancies.

## 2 Materials and methods

### 2.1 Animal rights statement

Experimental treatment regimens have been accomplished in compliance with the Iran's Animal Ethics system under the oversight of the Iran's community for the avoidance of harshness to Animals and Shiraz University study board (IACUC no: 4687/63). The recommendations of the EU Committee Directive (2010/63/EU) of September 22, 2010, on animal welfare standards for trial basis, were monitored as well.

### 2.2 Animals and fetal fluids collection

Seventeen pregnant domestic short-hair queens and nineteen pregnant mixed-breed bitches were submitted to the Veterinary Hospital of the School of Veterinary Medicine of Shiraz University for a population control program. The dogs and cats were owned and maintained in the School of Veterinary Medicine Shiraz University, ovariohysterectomized upon completion of the study, and retain in a non-government shelter. After a physical examination and approval of the animals` general health, gestational age was determined by measuring the gestational sac and biparietal head diameter according to a previous study (Bigliardi et al., 2022). Seventeen pregnant queens were aligned into days 30–39 (*n* = 5), 40–49 (*n* = 6) and 50–60 (*n* = 6) groups and 19 pregnant bitches were divided into days 30–39 (*n* = 6), 40–49 (*n* = 6) and 50–60 (*n* = 7) groups according to gestational age. Ketamine and midazolam (5 mg/kg and 0.2 mg/kg, respectively, IM) were used as pre-medication. Anesthesia induction was performed with propofol (4 mg/kg, IV) and then maintained by isoflurane (2 %) in oxygen. After the ovariohysterectomy, the gestational were sacs exposed and prepared for sampling. All fetuses were dead at the time of fetal fluid collection. The number of gestation sacs in each uterus was 8–11. Amniotic and allantoic sampling was performed from all gestation sacs in each uterus to measure APPs levels in different stages of pregnancy. The number of samples for each stages of pregnancy and for amniotic or allantoic fluid was at least five. The uterus was then carefully dissected, and gestational sacs were extracted completely. The allantoic fluid was first completely aspirated then the amniotic fluid collected. The allantoic and amniotic fluids were aspirated using separate 23 G needles connected to a 5 ml syringe, and then poured into a 1.5 ml microtube. The samples were promptly frozen and stored at -23 °C until the measurement of APPs.

### 2.3 Acute phase proteins measurement

Three reference samples at low, medium and high concentrations were used to determined the assay CVs. CRP was measured by a canine solid-phase sandwich ELISA method (Shanghai Crystal Day Biotech Company, China; Catalog Number: CRP E 0124ca) specified with intra-assay CV < 8 %, inter-assay CV < 10 %, and a sensitivity of 7.8 pg/ml. SAA was measured by a canine solid-phase sandwich ELISA method (Shanghai Crystal Day Biotech Company, China; Catalog Number: SAA E0125ca) specified with intra-assay CV < 8 %, inter-assay CV < 10 %, and a sensitivity of 0.156 pg/ml (Rajabi et al., 2023). AGP was measured by a commercial canine solid-phase sandwich ELISA kit (Immunology Consultants Laboratory Inc., Portland, OR, USA) with a sensitivity of 0.5 ng/ml, inter-assay CV < 10 %, and intra-assay CV < 8 % (Salavati et al., 2023).

### 2.4 Statistical analysis

GraphPad Prism7 was used for statistical analysis. The mean concentrations of CRP, SAA, and AGP were compared between different days of pregnancy and between the allantoic and amniotic fluids. Two-way ANOVA and Posthoc test were used for analysis. The significance level was set at *P* *<* *0.05*. Data were presented as mean ± SD.

## 3 Results

### 3.1 Dog

**Fig. 1:**
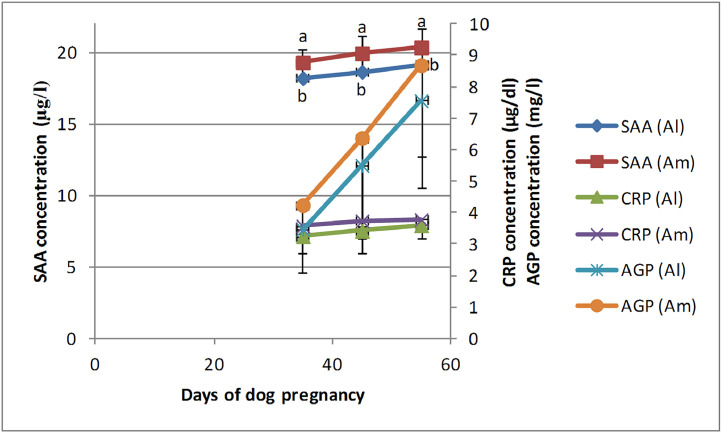
Changes and comparison of SAA, CRP, and AGP concentrations during canine pregnancy (days 30–39, 40–49, and 50–60) between the allantoic and amniotic fluids. ^ab^ Different letters denote significant differences between days of pregnancy.

The highest concentration of APPs in the allantoic and amniotic fluids was observed at the end of pregnancy during days 50–60. Although there was an increasing trend in the concentration of APPs, especially SAA and AGP, during the study days, these changes were not statistically significant (Fig. 1). Amniotic SAA concentration was significantly (*P* = 0.002) higher than that of allantoic. No significant difference was observed in the concentration of CRP and AGP between the allantoic and amniotic fluids during the study days (Fig. 1, Table 1).

**Table 1:** SAA, CRP, and AGP concentration (Mean± SD) during days of pregnancy in the canine and feline fetal fluids.

Dog						
	Allantoic fluid			Amniotic fluid		
**Days of pregnancy**	30–39	40–49	50–60	30–39	40–49	50–60
**SAA (µg/l)**	18.2 ± 1.02*	18.64 ± 1.32*	19.14 ± 1.31*	19.3 ± 0.89b*	19.94 ± 1.22*	20.36 ± 1.35*
**CRP (µg/dl)**	3.25 ± 0.16	3.42 ± 0.25	3.6 ± 0.40	3.57 ± 0.23	3.74 ± 0.23	3.78 ± 0.35
**AGP (mg/l)**	3.46 ± 1.35	5.5 ± 2.78	7.55 ± 2.77	4.24 ± 1.51	6.37 ± 2.82	8.68 ± 2.90
**Cat**						
	**Allantoic fluid**			**Amniotic fluid**		
**Days of pregnancy**	30–39	40–49	50–60	30–39	40–49	50–60
**SAA (µg/l)**	6.08 ± 2.05^a^	11.24 ± 4.34^b^	10.42 ± 4.65^ab^	6.48 ± 2.16	11.6 ± 4.33	10.87 ± 4.91
**CRP (µg/dl)**	15.04 ± 1.56	16.24 ± 1.11	16 ± 1.72	15.98 ± 1.54	17.22 ± 1	16.95 ± 1.53
**AGP (mg/l)**	13.6 ± 3.91	17.6 ± 5.12	19.5 ± 5.22	15.8 ± 4.32^a^	20.4 ± 5.02^ab^	22.25 ± 5.21^b^

⁎ Denote significant difference between canine allantoic and amniotic SAA concentration.

ab Different letters denote significant differences between pregnancy days for feline allantoic SAA and amniotic AGP.

### 3.2 Cat

**Fig. 2:**
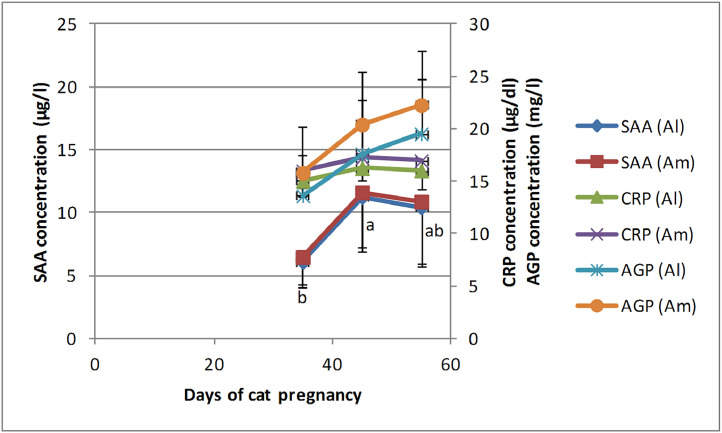
Changes and comparison of SAA, CRP, and AGP concentrations during feline pregnancy (days 30–39, 40–49, and 50–60) between the allantoic and amniotic fluids. ^ab^ Different letters denote significant differences between days of pregnancy.

The highest concentration of SAA and CRP in the allantoic and amniotic fluid was observed between days 40–49, while the maximum concentration of AGP was observed between days 50–60. Allantoic SAA concentration between days 40–49 was higher than between days 30–39 (*P* = 0.03). Similarly, amniotic AGP concentration between days 50–60 was higher than between days 30–39 (*P* = 0.04). In other cases, an increasing trend in the concentration of APPs in the allantoic and amniotic fluids was observed during the study, but the differences between days were not significant (Fig. 2). Furthermore, no significant changes in the concentration of APPs between the allantoic and amniotic fluids were observed during the study days (Fig. 2, Table 1).

## 4 Discussion

To the author`s knowledge, there is no data about the concentration of small carnivores` fetal fluid APPs. This study was therefore carried out to reveal the undiscovered aspects of fetal fluid. In the current study, the concentration of APPs, including SAA, CRP, and AGP in canine amniotic and allantoic fluid, increased during the pregnancy, and the maximum values were observed between days 50–60. However, the difference between days was not significant, and apart from the difference in SAA concentration (which was higher in amniotic fluid) between days 40–50 and 50–60, no significant difference was observed between the two fluids. APPs are mainly produced by hepatocytes, and considering the very low concentration of alanine aminotransferase and aspartate aminotransferase in canine fetal fluids (which may reflect liver immaturity) (Veronesi et al., 2018), maternal blood filtration is probably the main source of APPs in fetal fluids. Maternal serum APPs concentration during pregnancy is not affected by litter size and fluctuations in sexual hormones (Ulutas et al., 2009; Vannucchi et al., 2002). Serum APPs concentration, including fibrinogen, haptoglobin, seromucoid, and α2-globulin in pregnant bitches increased during gestation, and ceruloplasmin concentration increased before parturition (Vannucchi et al., 2002). These results could explain the increasing trend of APPs concentration in fetal fluids. Similarly, goats showed a significant increase in serum SAA concentration during the second month of gestation, which persisted elevated towards the end of pregnancy, peaking at parturition (Czopowicz et al., 2017).

APP concentrations increased in feline fetal fluids, but significant differences were observed in allantoic SAA at days 40–50 versus days 30–40 and in amniotic AGP concentration at days 50–60 versus days 30–40. There was no significant difference between the two fluids. The concentration of SAA in pregnant domestic cats increased until the mid-term and then decreased towards the end of pregnancy. In contrast to dogs, litter size positively affects SAA concentration (Naidenko et al., 2022).

CRP with a pentameric structure can combine to polysaccharide segment of parasite, bacteria and fungi while acting as an opsonin, trigger complement response and phagocytosis by monocytes and macrophages. SAA is in combination with lipoproteins and participate in innate immune response while CRP take part in adaptive immune system. In species that produce both CRP and SAA, studies have shown that the levels of SAA often rise and fall in a manner that mirrors the changes in CRP levels. This correlation can be useful in understanding the dynamics of the inflammatory response and can help in clinical assessments of disease states (Cray, 2012).

In felines, SAA is the acute phase protein that shows the most significant increase during inflammatory responses. This is followed by AGP and haptoglobin. Interestingly, unlike dogs where CRP is a prominent APP, it does not express the same level of response in cats. AGP is found in the seromucoid portion of serum and is primarily synthesized by hepatocytes during an acute phase response and is used to monitor inflammatory and neoplastic diseases in cats (Rosa & Mestrinho, 2019a).

There are various accumulation mechanisms for the volume and composition of fetal fluids in different animals at different gestational age that are not well understood. It is thought that dogs and cats have poorly vascularized amnion membrane which do not allow in-depth diffusion from and into it, so this feature make these fluids different but studies on feline fetal fluids revealed more similarity between these fluids proposing easier diffusion, this may explain higher amniotic SAA concentration in dogs and similar concentration of APPs in cats in this study(Tal et al., 2022).

Many unknown aspects of fetal fluids production, accumulation, absorption and diffusion especially during last stage of pregnancy make it difficult to evaluate the changes in the concentration of APPs between different days, for example at the end of pregnancy by urethral formation and urachus occlusion, urine is sent into amniotic fluid and the volume of fetal fluids will change (Sahraei et al., 2024). Further work on this topic with a larger population of healthy and pathologic pregnant dogs and cats is required to perceive differences and identify potential biomarkers for early detection of abnormalities.

## 5 Conclusions

The present study provides the first assessment of the acute phase proteins profile of canine and feline fetal fluids during the second half of pregnancy. The results of the current study pave the way for further investigations in this context. The absence of fetal fluid samples from the first half of pregnancy limited the results of this study. It is suggested to perform studies regarding measuring APPs in serum and fetal fluids in all stages of pregnancy in both normal and abnormal pregnant dogs and cats using both allantocentesis and amniocentesis.

## Declarations

### Ethics approval and consent to participate

Experimental treatment regimens have been accomplished in compliance with the Iran's Animal Ethics system under the oversight of the Iran's community for the avoidance of harshness to Animals and Shiraz University study board (IACUC no: 4687/63). The recommendations of the EU Committee Directive (2010/63/EU) of September 22, 2010, on animal welfare standards for trial basis, were monitored as well.

### Consent for publication

All authors approved the final article.

### Availability of data and materials

The datasets used and/or analyzed during the current study are available from the corresponding author on reasonable request.

## Funding

This study was supported financially by the Research Council of Shiraz University and School of Veterinary Medicine (Grant No. 1GCB4M198609)**.**

## CRediT authorship contribution statement

**Hossein Sahraei:** Writing – review & editing, Writing – original draft, Methodology, Investigation, Data curation, Conceptualization. **Asghar Mogheiseh:** Writing – review & editing, Writing – original draft, Visualization, Validation, Supervision, Software, Resources, Project administration, Methodology, Investigation, Funding acquisition, Formal analysis, Data curation, Conceptualization. **Fatemeh Doudmani:** Visualization, Methodology, Investigation. **Nasrin Kazemipour:** Validation, Methodology, Formal analysis, Data curation. **Saeed Nazifi:** Writing – original draft, Validation, Resources, Methodology, Investigation, Formal analysis, Data curation, Conceptualization.

## Declaration of competing interest

We would like to confirm that there are no known conflicts of interest associated with this publication and there has been no significant financial support for this work that could have influenced its outcome.
